# The Effects of a New Wireless Non-Adhesive Cardiorespiratory Monitoring Device on the Skin Conditions of Preterm Infants

**DOI:** 10.3390/s24041258

**Published:** 2024-02-16

**Authors:** Carmen M. Lorente Flores, Zhuozhao Zhan, Anouk W. J. Scholten, Gerard J. Hutten, Marieke Vervoorn, Hendrik J. Niemarkt

**Affiliations:** 1 Máxima Medical Center, Department of Neonatology, De Run 4600, 5504 DB Veldhoven, The Netherlands; carmen.lorenteflores@mmc.nl (C.M.L.F.); m.vervoorn@mmc.nl (M.V.); 2 Department of Mathematics and Computer Science, Eindhoven University of Technology, Groene Loper 3, 5612 AE Eindhoven, The Netherlands; z.zhan@tue.nl; 3 Department of Neonatology, UMC location University of Amsterdam, Meibergdreef 9, 1105 AZ Amsterdam, The Netherlandsg.j.hutten@amsterdamumc.nl (G.J.H.); 4 Amsterdam Reproduction & Development Research Institute, Meibergdreef 9, 1105 AZ Amsterdam, The Netherlands; 5 Department of Electrical Engineering, Eindhoven University of Technology, Groene Loper 3, 5612 AE Eindhoven, The Netherlands

**Keywords:** cardiorespiratory monitoring, wireless, skin damage

## Abstract

Aim: The aim of our study was to investigate skin conditions when wearing and removing a novel wireless non-adhesive cardiorespiratory monitoring device for neonates (Bambi-Belt) compared to standard adhesive electrodes. Study Design: This was a prospective study including preterm neonates requiring cardiorespiratory monitoring. Besides standard electrodes, the infants wore a Bambi Belt for 10 consecutive days. Their skin conditions were assessed using Trans Epidermal Water Loss (TEWL) and the Neonatal Skin Condition Score (NSCS) after daily belt and standard electrode removal. The ∆TEWL was calculated as the difference between the TEWL at the device’s location (Bambi-Belt/standard electrode) and the adjacent control skin location, with a higher ∆TEWL indicating skin damage. Results: A total of 15 infants (gestational age (GA): 24.1–35.6 wk) were analyzed. The ΔTEWL significantly increased directly after electrode removal (10.95 ± 9.98 g/m^2^/h) compared to belt removal (5.18 ± 6.71 g/m^2^/h; F: 8.73, *p* = 0.004) and after the washout period (3.72 ± 5.46 g/m^2^/h vs. 1.86 ± 3.35 g/m^2^/h; F: 2.84, *p* = 0.09), although the latter did not reach statistical significance. The TEWL was not influenced by prolonged belt wearing. No significant differences in the NSCS score were found between the belt and electrode (OR: 0.69, 95% CI [0.17, 2.88], *p* = 0.6). Conclusion: A new wireless non-adhesive device for neonatal cardiorespiratory monitoring was well tolerated in preterm infants and may be less damaging during prolonged wearing.

## 1. Introduction

In the clinical care of newborns, cardiorespiratory monitoring is often required to assess their clinical conditions and to detect and intervene on cardiorespiratory events in order to prevent morbidity and mortality. Classically, this is performed by electrocardiography (ECG) (for heart rate) and chest impedance (respiratory rate) using three wired adhesive electrodes [1].

The wires attached to the electrodes may restrict movement and form a hindrance in clinical care and—more importantly—Kangaroo Mother Care (KMC). As KMC is associated with improved survival and long-term outcomes and is therefore advised by the World Health Organization (WHO), any possible obstruction of KMC should be avoided [2,3].

Moreover, the adhesiveness of conventional electrodes may cause iatrogenic damage. This is particularly important in preterm infants as their immature skin is characterized by a thinner stratum corneum, which is the outermost layer of the dermis and plays an important role in the prevention of infection and heat and water loss [4]. Indeed, medical adhesive-related injuries (MARSIs) are the largest contributors to skin injury in the neonatal population [5], especially in preterm infants [6]. MARSIs lead to discomfort and pain, which are associated with impaired long-term neurocognitive outcomes in preterm infants, and increase the risk of sepsis [7].

The assessment of neonatal skin condition is a routine clinical practice [8,9] and can be performed with different visual skin assessment tools, such as the Neonatal Skin Condition Score (NSCS), which is a standardized and validated tool for assessing macroscopic skin damage [10]. However, MARSIs often consist of superficial or even microscopic damage, which is reflected by poor stratum corneum integrity. Although MARSIs may not be detectable by visual inspection, they can have deleterious consequences for the preterm infant, such as water loss, electrolyte imbalance, and infection. The loss of stratum corneum integrity can be demonstrated by assessing the Trans Epidermal Water Loss (TEWL). Therefore, although not part of daily clinical practice, the most common method to thoroughly assess skin condition and damage is by the measurement of the TEWL. This can be performed swiftly and non-invasively at the bedside by using a Vapometer [7,11,12,13]. In multiple studies, the removal of adhesive electrodes and medical tapes has been shown to be associated with increased TEWL [14].

To avoid the hindrance of wires and skin injury, a novel wireless non-adhesive sensor belt (Bambi Belt, Bambi Medical, Eindhoven, the Netherlands) has been developed for cardiorespiratory monitoring. The feasibility and safety of this device were tested in a pilot study recently, with the measurements lasting up to three days [15]. Furthermore, the belt was non-inferior in heart rate monitoring compared to standard ECG with adhesive electrodes [16]. Nevertheless, the effects of prolonged Bambi Belt wearing on skin conditions have not been investigated.

The aim of this study was to investigate the effects of prolonged wear and removal of the Bambi Belt on skin conditions in preterm infants compared to the standard adhesive electrode, as measured by the TEWL and NSCS. Additionally, we investigated whether the duration of belt wearing and skin maturation influenced the outcomes. 

## 2. Materials and Methods

### 2.1. Study Design and Recruitment

This study was performed in Máxima Medical Center Veldhoven, the Netherlands, from July 2021 to January 2022. This study was approved by the Ethical Board of the Máxima Medical Centre (NL77561.015.21) and registered in the Netherlands Trial Register (NL 9478). This study was performed in addition to a multicenter monitor performance study of the Bambi Belt (NL9480) [16]. This study was monitored by an independent body (Clinical Trial Centre Maastricht). This study was partly funded by Bambi Medical B.V.

**Figure 1:**
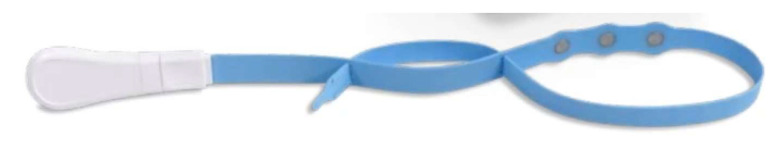
Bambi Belt used in clinical practice.

Parents of newborn infants born in the Maxima Medical Centre Veldhoven and included in the multicenter monitor performance trial (requiring long-term cardiorespiratory monitoring) were approached (see Figure 1). Infants with visible skin lesions prior to the study, major congenital anomalies, or expected surgery or MRI (which prevented the use of the Bambi Belt) were excluded. To obtain a representative sample (and to compensate for the fact that parents of older stable neonates are more inclined to give consent), participants in the monitor study were included in three postmenstrual age (PMA) strata: <28 wk; 28–32 wk; and >32 wk.

Included infants were monitored with the standard monitoring system (Intellivue MP90, Philips Healthcare, Eindhoven, the Netherlands) with adhesive electrodes (3M™ Red Dot™ or Ambu^®^ Blue Sensor NEOX) and simultaneously wore the Bambi Belt (Bambi Medical, Eindhoven, the Netherlands) for 10 consecutive days. Clinical care was provided as usual.

During the study period, the Bambi Belt was removed shortly and routinely at least once a day, e.g., during weighing. At this moment, the skin condition was assessed (if the patient’s medical condition allowed it). When the belt was removed more than one time a day, the skin assessment was only performed once. For the standard adhesive electrodes, no scheduled moment was defined for removal. They were only removed when it was deemed clinically necessary, and skin condition was only assessed at this time.

Skin assessment was performed in 2 ways: First, the TEWL was measured as primary endpoint with use of a Vapometer (Delfin Technologies Ltd., Kuopio, Finland). This validated and calibrated device provides a non-invasive and quick (10–20 s) TEWL measurement after gently holding it against the skin on the desired location [13]. TEWL measurement was standardized and consisted of four sub-measurements: two measurements at the belt/adhesive electrode location, and two measurements of the skin adjacent to the belt/electrode (control). TEWL measurements took place directly after removal of the belt and after a washout period of 3 to 5 min. ∆TEWL was calculated as the difference between TEWL on the belt/electrode location and the control location. 

Second, skin condition was assessed using the Neonatal Skin Condition Score (NSCS), a clinically validated visual observation scale [10], which is used in daily clinical practice. It consists of a 9-point scale addressing skin dryness, erythema, and skin breakdown or excoriation to assess skin condition. Scores can vary from 3 (normal) to 9 (worst) (see Table 1). NSCS and TEWL values were logged in the electronic case report form together with clinical and demographic data and information about room temperature, humidity, and clothing.

### 2.2. Investigational Device

The Bambi Belt is a disposable device for wireless cardiorespiratory monitoring of preterm infants in a hospital environment. It is made of a soft stretchable material with integrated dry electrodes that are able to measure the electrical activity of the diaphragm. It is wrapped around the chest of the infant without the need for skin preparation. It is designed to be skin-friendly and to allow for multiple removals during the day, for instance, when patients are weighed, bathed, or examined. To accommodate for the entire neonatal population, the belt is available in four different sizes, which all cover a range of chest sizes due to a gradual closing mechanism on one end. The other end holds a connector as a click-on site for the sensor module (Figure 1). 

### 2.3. Sample Size Calculation

A sample size of 15 infants, at the one-sided 5% significance level, was determined to provide 80% power to detect a mean difference of 5 g/m^2^/h and a standard deviation of 5 between the paired measurements of ∆TEWL of the two different devices [14], as based on Wilcoxon’s signed rank test. The latter is a (more conservative than a parametric) test, chosen based on the expected inter-patient variability. 

### 2.4. Data Analysis

As a primary outcome, we assessed differences in ∆TEWL. First, we assessed differences in ∆TEWL between the Bambi Belt and adhesive electrode removals directly after removal and after the washout period. Second, to assess the toleration of prolonged Bambi Belt wear, we assessed whether the ∆TEWL for belt removal changed with the increasing study day (i.e., prolonged belt wearing and increased removals). For both analyses, we created mixed-effect analysis of variance (ANOVA) models of the ∆TEWL with different devices (Bambi Belt/adhesive) and on all study days. The device and study day were both considered as fixed effects in the ANOVA model, while patient characteristics (gestational age (GA), humidity, temperature, and clothing) were considered as random effects. Statistical significance of the fixed effects was tested using a Type III F-test at a significance level of 0.05 with the Satterthwaite approximated denominator degrees of freedom. 

To assess whether skin maturity influences Bambi Belt toleration, a linear mixed-effect model (LMM) with an infant-specific random intercept was fitted to the absolute TEWL values with the GA, the study day, and their interactions included as fixed effects, and room temperature, humidity, and clothing of the infant included as random effects.

At last, the overall NSCS scores were investigated between the two devices. Since the total score is calculated on a Likert scale, an ordinal cumulative logit mixed-effect model was used to investigate if the risk of having a higher NSCS score was different between the two devices. 

All statistical analyses were performed using R version 4.0 (the R Foundation for Statistical Computing; Vienna, Austria) and SAS software version 9.4 (SAS Institute Inc., Cary, NC, USA). Note that, based on the primary outcome, infants were excluded from analysis when the study terminated before 10 days without having had adhesive electrodes removed. Data from these excluded patients were assessed for outliers or noteworthy TEWL values. 

## 3. Results

**Figure 2:**
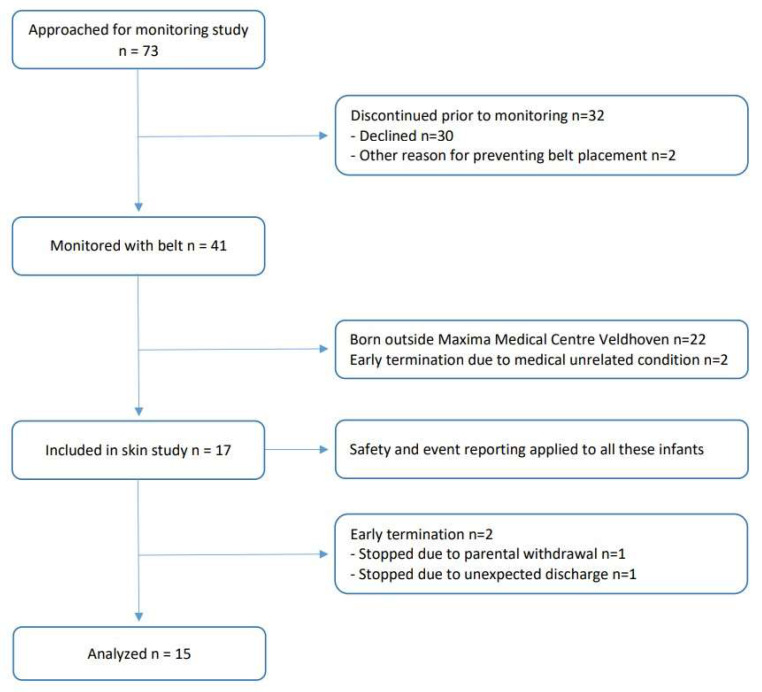
Study flow chart. Infants admitted to the neonatal wards requiring long-term cardiorespiratory monitoring were eligible. In total, 17 infants were recruited, and 2 infants were excluded from the analysis because the adhesive electrodes were removed before the end of the 10-day study period (1 due to unforeseen discharge and 1 due to withdrawal of consent). As such, 15 infants were analyzed.

In total, 17 infants were recruited (see flowchart in Figure 2). Two infants were excluded from the analysis because the adhesive electrodes were removed before the end of the 10-day study period (one was excluded due to unforeseen discharge, and one was excluded due to withdrawal of consent). As such, 15 infants with a median GA of 28.0 weeks (IQR: 26.3–32.0) and a median PMA of 28.9 weeks (IQR: 27.4–33.3) at the start of the study were analyzed (for demographic information, see Table 2), yielding 132 Bambi Belt removal assessments and 13 adhesive removal assessments throughout the study. In two infants, the adhesive electrodes were not removed during the whole 10-day study period.

Table 3 shows the absolute TEWL and ∆TEWL values. The mixed-effect ANOVA model showed that the ΔTEWL was significantly higher after adhesive electrode removal (10.95 (±9.98) g/m^2^/h) compared to the Bambi Belt removal (5.18 ± 6.72 g/m^2^/h; F-value: 8.73, *p* = 0.004). After the washout period, the ∆TEWL still remained twice as high for the electrodes (3.71 ± 5.18 g/m^2^/h) compared to that of the Bambi Belt (1.86 ± 3.35 g/m^2^/h), but this difference was not statistically significant (F-value: 2.84, *p* = 0.09). 

The study day did not significantly affect the ∆TEWL results directly after the belt removal (F-value: 1.81, *p*-value: 0.06) and after the washout period (F-value: 1.67, *p*-value: 0.09).

When assessing the absolute TEWL values after correcting for temperature, humidity, and clothes that the infants wore, a significant effect was found for the GA, but not for the study day, at both the belt and control locations (Table 4). Furthermore, we could not demonstrate an interaction between the GA and the study day on the TEWL at the belt nor control locations.

In Table 5, the NSCS scores are provided. After the removal of both the Bambi Belt and adhesive electrode, NSCS scores above >3 were found in 19.7% and 23.1% of cases, respectively. No significant differences in the odds ratios for higher NSCS scores were found after belt removal versus adhesive electrode removal (OR: 0.69, 95% CI: [0.17, 2.88], *p*-value: 0.6). In three instances, redness of the skin was reported as an adverse event, of which two instances were related to the belt and one to the adhesive electrodes.

In the secondary analysis, the data from the patients that were excluded from analysis (n = 2; see Figure 2) were comparable to the data from all other patients, with an immediate TEWL value of 14.03 g/m^2^/h and a washout TEWL value of 10.93 g/m^2^/h at the belt location.

## 4. Discussion

In this study, we investigated the effects of a new wireless non-adhesive belt (Bambi Belt) for neonatal cardiorespiratory monitoring of skin conditions compared to standard adhesive electrodes. Skin conditions were assessed by measuring the TEWL and NSCS score. The increase in TEWL after belt removal was significantly lower than that after the removal of the adhesive electrodes, indicating less microscopic skin damage. Moreover, prolonged Bambi Belt wear and repeated removals did not influence the ΔTEWL, demonstrating that the belt was well tolerated if applied for at least ten days. Toleration for prolonged use was not influenced by the GA. The NSCS, as an indicator of macroscopic skin damage, did not show a statistical difference between the Bambi Belt and adhesive electrodes. 

TEWL reflects the stratum corneum integrity and therefore indicates both skin maturation (as the stratum corneum increases with maturation) and skin damage [17,18]. Indeed, we demonstrated that TEWL reduced with the increasing PMA. However, as a comparison (between Bambi Belt and adhesive electrodes) took place at the same time points within the same patients, the effect of skin maturation on the primary outcome was considered negligible. To assess if prolonged belt wear affects skin maturation, we performed a multilevel analysis with the GA and study day as inputs. The maturational effect on the TEWL was similar between the belt and control locations. The skin is an important part of the body acting as a sensory organ and as a barrier for water loss and infection. Even superficial damage to fragile preterm skin may have consequences, such as pain, infection, and water loss. These may impose long-term neurodevelopmental consequences for preterm infants [19,20]. In addition to avoiding the hindrance of wires, the investigated Bambi Belt prevents the loss of skin integrity and may therefore be a skin-friendly solution for cardiorespiratory monitoring and contribute to improving neonatal care for this vulnerable population. To our knowledge, the Bambi Belt is the first wireless device for neonatal cardiorespiratory monitoring that has been clinically tested for long-term skin compatibility.

We did not find significant differences in the NSCS scores, probably due to the low sample size. However, we considered the TEWL to be a more objective and quantitative measure for skin integrity that also detects more subtle skin damage. Therefore, we specified the TEWL for the primary outcome and sample size calculation [13,21,22]. In order to detect differences in the NSCS scores, a larger implementation study has to be performed in the future.

During the study, three non-serious adverse skin events were reported, of which two were related to the belt (redness of the skin at belt location). Because of the fugacious nature, no medical interventions were warranted in any of these cases. In both cases involving the belt, the study was stopped upon the request of the parents. In one of these patients, the adhesive electrodes were not removed, and therefore, a comparison between the adhesive electrodes and the belt could not take place. This patient was excluded from the analysis. 

An in-depth evaluation of these two belt-related adverse events showed that these were associated with the malposition of the belt, which had shifted and/or twisted, resulting in the loss of contact of the electrode with the skin. Due to the study’s design, the monitoring and alarming function was disabled during prolonged wearing, and the nursing staff were not directly alerted to signal loss due to belt mispositioning. Therefore, the belt was not immediately repositioned. In our opinion, a swift replacement of the belt to correct the position would have prevented these adverse events. Indeed, in our 24 h feasibility and monitoring study, no adverse skin events were reported [15,16].

As we excluded infants with visible skin lesions, caution must be taken when using the belt in infants with clinically very fragile skin, such as newly born, extremely preterm infants with a postmenstrual age of <26 weeks or in infants in which medical skin conditions are suspected.

Most skin assessments took place during morning rounds, when the belt was removed for weighing or when it was decided that cardiorespiratory monitoring using adhesive electrodes was no longer necessary. Therefore, circadian effects on skin condition were deemed irrelevant to our results. 

As skin lesions can develop over time, we arbitrarily decided on a 10-day study period. In order to avoid methodological issues with a longer study period, such as the disappearance of the need for cardiorespiratory monitoring or transfer to another ward, we did not choose a longer period. Future implementation studies should investigate the effects of wearing the belt during the whole stay in the ward.

Nevertheless, this study has some limitations. For instance, although we had a well-designed sample size, the sample size was small, and blinding was not possible due to the study’s design. Nevertheless, it is of great importance to comply with targeted sample size calculations when performing a 10-day study with an extra medical device in this extremely vulnerable population where studies are, by nature, a burden. Future implementation studies comparing infants wearing a functional Bambi Belt versus infants wearing conventional adhesive electrodes should confirm our results. Furthermore, as this study was performed in a vulnerable population, it was deemed unethical to remove the adhesive electrodes daily for TEWL measurements when this was not clinically necessary. This led to a difference in the observations of adhesive electrode removal and Bambi Belt removal. However, the persistent effects of adhesive electrode removal on microscopic skin damage have been extensively described [23,24,25,26], and the findings in our study are in close agreement with the results of previous studies.

## 5. Conclusions

The prolonged wear of a new wireless and non-adhesive belt for cardiorespiratory monitoring was associated with more favorable effects on skin conditions in preterm infants compared to the use of classic adhesive electrodes. This belt may offer a skin-friendly alternative for cardiorespiratory monitoring in this vulnerable population. 

## Figures and Tables

**Table 1:** Neonatal Skin Condition Score (NSCS).

Dryness	Erythema	Breakdown/Excoriation
1. Normal, no signs of dry skin	1. No evidence of erythema	1. Non evident
2. Dry skin, visible scaling	2. Visible erythema <50% of body surface	2. Small localized areas
3. Very dry skin, cracking/fissures	3. Visible erythema >50% of body surface	3. Extensive

**Table 2:** Subject demographics. Median and IQR are shown for different variables.

Demographics	
Gestational age, GA (wk)	28 [IQR: 26.3–32.0]
Birth weight (grams)	1040 [IQR: 880–1550] 4/11
Gender (F/M)	4 (26.7%)/11 (63.3%)
Postmenstrual age at start of study (days)	9 [IQR: 6–12]
Respiratory support at start of study	
NIPPV/SIPPV	2 (13.3%)
NCPAP/duoPAP	8 (53.3%)
High/Low flow	1 (6.7%)
None	4 (26.7%)
Phototherapy during study (with LED lamp)	2 (13.3%)

**Table 3:** Summary for Trans Epidermal Water Loss (TEWL) measurements. ∆TEWL was calculated as TEWL at belt location-TEWL at control location for each measurement.

Device	∆TEWL (g/m^2^/h)		TEWL Device Location (g/m^2^/h)		TEWL Control Location (g/m^2^/h)	
	Immediate Mean (SD)	Washout Mean (SD)	Immediate Mean (SD)	Washout Mean (SD)	Immediate Mean (SD)	Washout Mean (SD)
Bambi Belt	5.18 (6.72)	1.86 (3.35)	14.43 (9.98)	10.76 (7.86)	9.26 (7.74)	8.89 (7.71)
Electrodes	10.95 (9.98)	3.72 (5.46)	20.87 (16.72)	14.03 (13.17)	9.92 (11.25)	10.32 (12.52)

**Table 4:** Influence of skin maturation (gestational age: GA) and prolonged wearing of belt (study day) on absolute TEWL measurements.

	Belt Location	Control Location
**GA (weeks)**	−2.44 SE: 1.16 *p*-value: 0.04	−2.05 SE: 1.00 *p*-value: 0.04
**Study day (days)**	−4.08 SE: 6.24 *p*-value: 0.51	−6.54 SE: 5.27 *p*-value 0.22
**Interaction GA × study day**	0.10 SE: 0.23 *p*-value: 0.66	0.22 SE: 0.19 *p*-value: 0.28

**Table 5:** Neonatal Skin Condition Scale (NSCS) evaluations in study population.

Total NSCS Score	Location (Frequency)					
	Bambi-Belt	%	Electrodes	%	Control Area	%
3	106	80.3	10	76.9	116	91.3
4	21	15.9	2	15.4	9	7.1
5	4	3.0	1	7.7	1	0.8
6	1	0.8	0	0	1	0.8
All	132	100	13	100	127	100

## Data Availability

The datasets generated during and/or analyzed during the current study are not publicly available so as to not compromise individual privacy, seeing that the data contain, for instance, age and maturation information needed to replicate the results. The data are available from the corresponding author upon reasonable request.
